# Dose-dependent effects of mTOR inhibition on weight and mitochondrial disease in mice

**DOI:** 10.3389/fgene.2015.00247

**Published:** 2015-07-22

**Authors:** Simon C. Johnson, Melana E. Yanos, Alessandro Bitto, Anthony Castanza, Arni Gagnidze, Brenda Gonzalez, Kanav Gupta, Jessica Hui, Conner Jarvie, Brittany M. Johnson, Nicolas Letexier, Lanny McCanta, Maya Sangesland, Oliver Tamis, Lauren Uhde, Alex Van Den Ende, Peter S. Rabinovitch, Yousin Suh, Matt Kaeberlein

**Affiliations:** ^1^Department of Pathology, University of WashingtonSeattle, WA, USA; ^2^Department of Genetics, Albert Einstein College of MedicineNew York, NY, USA; ^3^Department of Psychology, University of WashingtonSeattle, WA, USA

**Keywords:** aging, rapamycin, mTOR, mitochondrial disease, pharmaceutical intervention

## Abstract

Rapamycin extends lifespan and attenuates age-related pathologies in mice when administered through diet at 14 parts per million (PPM). Recently, we reported that daily intraperitoneal injection of rapamycin at 8 mg/kg attenuates mitochondrial disease symptoms and progression in the *Ndufs4* knockout mouse model of Leigh Syndrome. Although rapamycin is a widely used pharmaceutical agent dosage has not been rigorously examined and no dose-response profile has been established. Given these observations we sought to determine if increased doses of oral rapamycin would result in more robust impact on mTOR driven parameters. To test this hypothesis, we compared the effects of dietary rapamycin at doses ranging from 14 to 378 PPM on developmental weight in control and *Ndufs4* knockout mice and on health and survival in the *Ndufs4* knockout model. High dose rapamycin was well tolerated, dramatically reduced weight gain during development, and overcame gender differences. The highest oral dose, approximately 27-times the dose shown to extend murine lifespan, increased survival in *Ndufs4* knockout mice similarly to daily rapamycin injection without observable adverse effects. These findings have broad implications for the effective use of rapamycin in murine studies and for the translational potential of rapamycin in the treatment of mitochondrial disease. This data, further supported by a comparison of available literature, suggests that 14 PPM dietary rapamycin is a sub-optimal dose for targeting mTOR systemically in mice. Our findings suggest that the role of mTOR in mammalian biology may be broadly underestimated when determined through treatment with rapamycin at commonly used doses.

## Introduction

Rapamycin, a specific inhibitor of the mechanistic Target Of Rapamycin (mTOR), was the first pharmacological agent reproducibly demonstrated to extend lifespan in multiple organisms including budding yeast, nematodes, flies, and mice (Harrison et al., 2009; Ha and Huh, 2011; Partridge et al., 2011; Choi et al., 2013; Johnson et al., 2013a,b). Rapamycin was first demonstrated to increase murine lifespan by the National Institute on Aging (NIA) Interventions Testing Program (ITP) in a study where genetically heterogeneous UMHET3 mice were fed a diet supplemented with 14 PPM rapamycin in a microencapsulated formula beginning at around 600 days of age (Harrison et al., 2009). This diet was subsequently shown to extend lifespan in UMHET3 mice treated from a young age (Miller et al., 2011) and C57Bl/6 inbred mice when initiated at either 19 months of age (Zhang et al., 2014) or mixed ages (Neff et al., 2013). Surprisingly, dietary treatment with rapamycin at 14 PPM had comparable effects on survival whether treatment was initiated in young or middle-aged animals. Similar to dietary delivery, an intermittent treatment protocol where 1.5 mg/kg rapamycin was injected subcutaneously three times per week, 2 weeks out of every month, was shown to extend lifespan in the 129/Sv background starting in young animals (Anisimov et al., 2011). In addition to increasing lifespan, there is a general consensus that rapamycin attenuates age-associated declines in some measures of cardiac, immune, muscular, and cognitive function, increasing overall healthspan (Spong and Bartke, 2012; Wilkinson et al., 2012; Blagosklonny, 2013; Kaeberlein, 2013; Neff et al., 2013; Zhang et al., 2014). mTOR inhibition by rapamycin has also been shown to provide benefit in models of disease such as the murine model of Leigh Syndrome.

Leigh Syndrome (LS) is a severe mitochondrial disease that occurs in about 1:40,000 newborns and is associated with retarded growth, muscular deficits including myopathy and dyspnea, lactic acidosis, and a characteristic progressive necrotizing encephalopathy of the vestibular nuclei, cerebellum, and olfactory bulb (Budde et al., 2002, 2003; Anderson et al., 2008). *Ndufs4* encodes a subunit of Complex I of the mitochondrial electron transport chain; mutations in the NDUFS4 gene cause LS in humans (Budde et al., 2000, 2003; Darin et al., 2001; Anderson et al., 2008; Quintana et al., 2010), and the *Ndufs4* knockout (KO) mouse is a murine model of LS (Kruse et al., 2008; Quintana et al., 2010). *Ndufs4* KO mice have decreased Complex I levels and activity in multiple tissues and show severe and progressive symptoms of mitochondrial disease that mirror human LS. LS results in death at an average of 6–7 years in humans, and *Ndufs4* KO mice show a similar early-life mortality with an average lifespan of just 50 days.

In a recently published study, we reported a striking suppression of Leigh Syndrome phenotypes by rapamycin in *Ndufs4* KO animals (Johnson et al., 2013c). 8 mg/kg of rapamycin increased survival by roughly 30% when administered every other day via intraperitoneal injection (IP) and more than doubled median survival and tripled maximum survival when administered every day. In addition to the robust effect on survival, daily injection of rapamycin also attenuated multiple aspects of disease including neuroinflammation, weight loss, and behavioral phenotypes associated with neurological decline. Given these findings, we sought to determine whether oral delivery of encapsulated rapamycin could achieve effects similar to daily IP injection in regards to developmental weight gain in wildtype mice and disease in *Ndufs4* KO animals. Here, we report that oral delivery of encapsulated rapamycin has dose-dependent effects on developmental weight in mice and disease onset and progression in the murine model of LS. A dosage of 378 PPM, approximately 27-fold higher than that shown to extend lifespan of WT animals, was necessary to achieve effects on survival and body composition in control and *Ndufs4* KO animals comparable to daily IP injection of 8 mg/kg rapamycin. Importantly, although these high-doses of rapamycin resulted in a dramatic reduction in animal size, reminiscent of pituitary dwarfism, we observed no detrimental effects in treated mice. These studies have major implications for experimental and clinical use of rapamycin.

## Materials and methods

### Animals

Heterozygous *Ndufs4* knockout mice on a C57Bl/6NIA background were bred to produce homozygous KO animals. Injectable rapamycin preparation and delivery and animal care and end-of-life criteria were as previously described (Johnson et al., 2013c). All animal use was in accordance with the University of Washington institutional guidelines and experiments were performed as approved by the Institutional Animal Care and Use Committee. *Ndufs4* heterozygous mice and wild-type mice are phenotypically indistinguishable in all studies (both here and previously published) and are pooled as controls for the experiments described. All mice used in these studies were C57Bl/6NIA animals.

Encapsulated rapamycin was obtained from the Barshop Institute at the University of Texas Health Science Center at San Antonio and Rapamycin Holdings, Inc. Standard mouse chow was ground to a powder and mixed with encapsulated rapamycin at 14, 42, 126, or 378 PPM. 300 mL of 1% agar melted in sterile water was added per kilogram of powdered chow and the mixture was pelleted and baked at 55°C for 2–3 h to harden. Pellets were then vacuum-sealed for short-term (4°C) or long-term (−20°C) storage. Control food contained either no drug or encapsulation material (eudragit) alone at a concentration matching that in the rapamycin chow, as indicated.

Mice began receiving assigned diet treatments upon weaning, at 20–21 days of age. Mice assigned to rapamycin (8 mg/kg) or vehicle injection groups began treatments at post-natal day 10 (P10) as previously described (Johnson et al., 2013c).

### Rapamycin blood level analysis

Whole-blood samples were collected in EDTA tubes by cardiac puncture immediately following euthanasia. All blood samples were collected at approximately the same time of day, during light cycle, to minimize variation between samples due to feeding. Samples were frozen and shipped on dry ice to the Javors laboratory in the Department of Psychiatry at the University of Texas Health Science Center San Antonio for analysis by HPLC-MS.

### Statistical analysis

**Figure 1:**
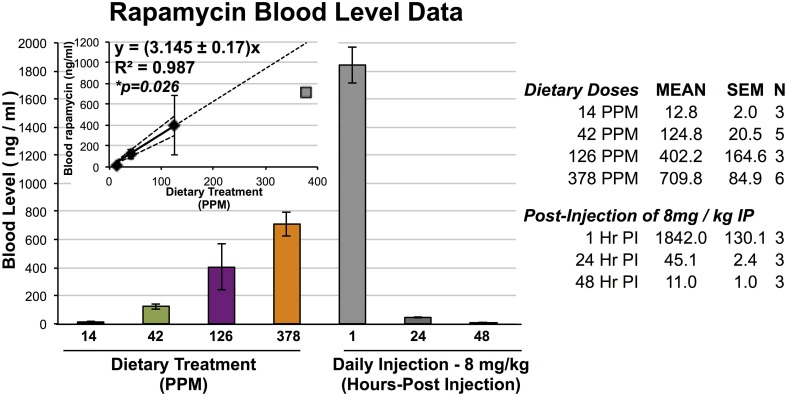
**Blood levels of rapamycin resulting from dietary and IP injection delivery**. Blood levels of rapamycin in mice treated with dietary rapamycin or intraperitoneal injection as indicated. Dietary concentration to blood level relationship shown in inlayed graph; linear regression generated using 14, 42, and 126 PPM data with 95% confidence intervals, correlation co-efficient, and correlation *p*-value indicated. All error bars represent SEM.

Linear regression and related statistics in Figure 1A were generated in GraphPad Prism using data for 14, 42, and 126 PPM rapamycin with y-intercept was set to 0. Survival curves were compared by Log-rank test in GraphPad. Pairwise *t*-tests were performed in Excel. All tests were two-tailed and assumed equality of variance, with a *p*-value less than 0.05 considered statistically significant.

## Results

### Dietary rapamycin reduces developmental weight in a dose-dependent manner

To assess the efficacy and effects of high-dose dietary rapamycin, control and *Ndfus4* KO animals were fed chow containing 42, 126, or 378 PPM rapamycin beginning at weaning (~P20, see Materials and Methods). Circulating levels of rapamycin were measured after at least 7 days on the rapamycin diet (Figure 1). Levels of circulating rapamycin increased in a linear manner from 14 to 126 PPM at a rate of approximately 3.2 ng/ml serum rapamycin per PPM dietary rapamycin increase, with linearity decaying between 126 and 378 PPM.

**Figure 2:**
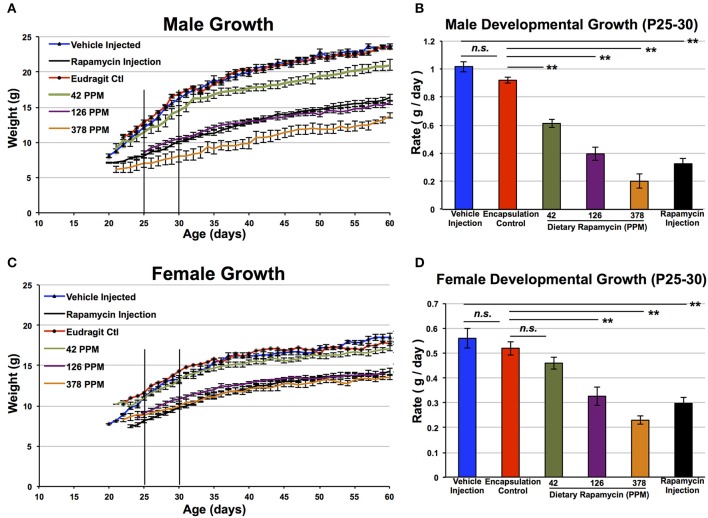
**Rapamycin reduces developmental weight gain in a dose-dependent manner**. Control male **(A,B)** and female **(C,D)** mice treated with dietary rapamycin from weaning (approximately post-natal day 21). Rate calculated from post-natal day 25–30 was reduced in a dose-dependent manner in both genders. Eudragit (encapsulation material) alone at a quantity equivalent to that in 378 PPM rapamycin chow had no impact on developmental weight gain. Male and female mice treated with 378 PPM dietary rapamycin show similar weight curves and developmental weight gain rates. Rapamycin injection—8 mg/kg/day IP injection. Error bars represent SEM. ^**^p < 0.005; n.s., not significant.

Dietary rapamycin reduced developmental weight gain in a dose-dependent manner in control animals, with strength of effect ranging from no observed impact at 14 PPM (data not shown), to a robust and highly significant reduction in body size at 378 PPM that is similar in magnitude to what we previously reported for daily injection of 8 mg/kg (Figure 2). Rate of weight gain during 25–30 days of life was decreased in a dose-dependent manner. Mice treated with the highest doses of rapamycin are approximately 50% the size of control littermates at 30 and 60 days of age, reminiscent of genetic interventions in growth signaling such as the long-lived Snell and Ames dwarf mice (Cheng et al., 1983). While gender specific effects of rapamycin at low doses are well established, we found that at 378 PPM rapamycin male and female animals have indistinguishable rates of weight increase. Although a robust decrease in developmental weight gain occurred in both genders by 378 PPM, it should be noted that this may not be the level of maximum possible effect as we did not examine higher doses.

### Dietary rapamycin increases Ndufs4 knockout survival in a dose-dependent manner

**Figure 3:**
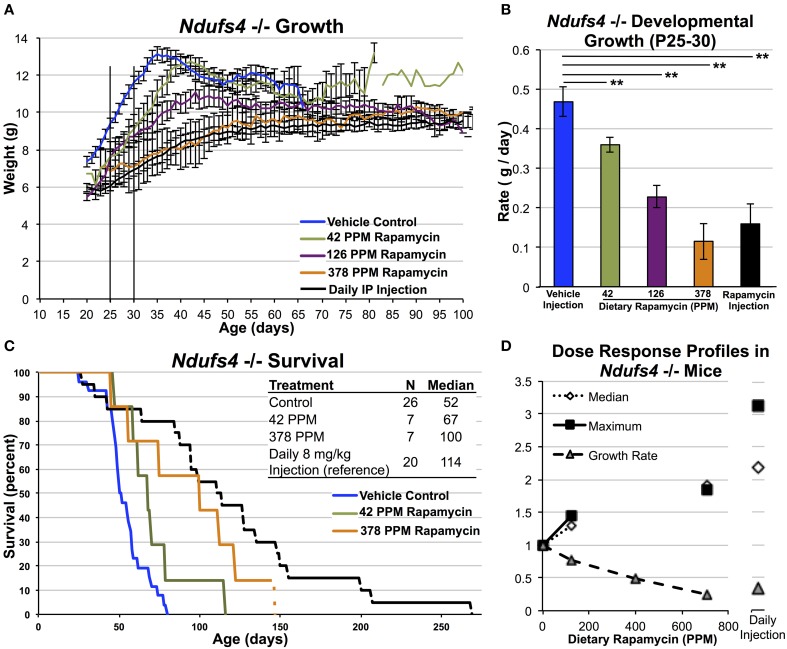
**High-dose dietary rapamycin attenuates disease and enhances survival in** ***Ndufs4*** **^−/−^ mice**. Dietary rapamycin reduces developmental weight gain and attenuates the progressive weight loss phenotype in a dose-dependent manner **(A,B)**. Dietary rapamycin improves survival in a dose-dependent manner **(C)**. The effect of 378 PPM dietary rapamycin on weight is slightly greater than daily IP rapamycin injection while IP injection resulted in a greater increase in median and maximum survival **(D)**. The oldest mouse in the 378 PPM group was euthanized due to lack of chow and was still healthy by gross observation at the time of euthanasia. Rapamycin injection—8 mg/kg/day IP injection. Error bars represent SEM. ^**^p < 0.005. 378, but not 42, PPM treatment significantly increased survival by Log-rank test (*p* = 0.086 and 0.006 for 42 and 378 PPM, respectively; IP injection shown for reference, previously reported elsewhere) (Johnson et al., 2013b).

We next assessed the impact of dietary rapamycin on *Ndufs4* KO animals fed a diet containing 42, 126, or 378 PPM rapamycin beginning at weaning. As previously shown, control treated *Ndufs4* KO mice show a slightly reduced developmental weight gain compared to control animals until 35 days of age where disease symptoms onset. This is followed by a progressive decline in body weight concomitant with the presentation of neurological symptoms and a steady loss of fat mass (Johnson et al., 2013c). We found that dietary rapamycin treatment altered weight profiles of *Ndufs4* KO animals in a dosage dependent manner similar to the effects in wildtype animals (Figure 3). 14 PPM dietary rapamycin had no impact on disease onset or progression (data not shown). 42 PPM dietary rapamycin delayed the rapid decline in weight roughly proportionally to the increase in survival for this cohort (Figures 3C,D), while mice treated with 126 PPM or 378 PPM showed a reduced rate of weight gain but were largely protected against the rapid weight loss occurring around P35 in control animals, showing similar weight profiles to *Ndufs4* KO animals treated with 8 mg/kg/day of rapamycin by IP injection. These observations are consistent with the effects of daily IP injection of 8 mg/kg rapamycin beginning around weaning and demonstrate that high doses of dietary rapamycin are well tolerated and are required in order to achieve robust benefits from the drug in this disease model.

A comparison of the effects of dietary rapamycin and daily rapamycin injection on median lifespan, maximum lifespan, and rate of developmental weight gain reveals that 378 PPM impacts developmental weight gain roughly equivalently to daily IP rapamycin at 8 mg/kg but may appear not to be sufficient to achieve the same benefits in regards to lifespan in the *Ndufs4* KO animals (Figure 3D, see discussion).

### Rapamycin treatment starting at P35 delays mortality without impacting developmental weight gain in the Ndufs4 ^−/−^ mice

**Figure 4:**
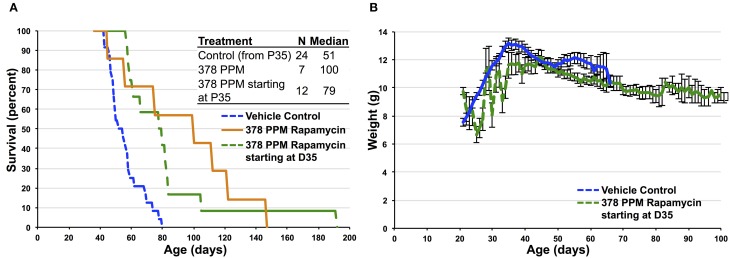
**Impact of high-dose dietary rapamycin on** ***Ndufs4*** **^−/−^ mice when initiated at P35**. **(A)** High-dose rapamycin provides some benefit to survival when initiated at P35 without impacting developmental weight **(B)**. Control animals in **(A)** only include those surviving past P35. Survival of mice treated starting at P35 is significantly greater than control treated animals (*p* = 0.0002, Log-rank test).

As most cases of severe mitochondrial disease in children are diagnosed after the presentation of symptoms, we sought to determine if rapamycin can have a beneficial impact on mitochondrial disease when initiated after the onset of neurological symptoms. To address this we treated a group of knockout animals with 378 PPM dietary rapamycin starting at P35, the age at which weight peaks in the *Ndufs4* knockout mice and neurological phenotypes become apparent. Animals in this cohort showed a significant delay in mortality relative to controls, although the rescue was not as robust as that of 378 PPM started at weaning (Figure 4). These data indicate that the benefits of rapamycin are at least partly independent of any effects the drug may have on very early developmental weight, but that earlier treatment provides the greatest benefit.

### New insight into the impact of rapamycin on aging and disease

**Figure 5:**
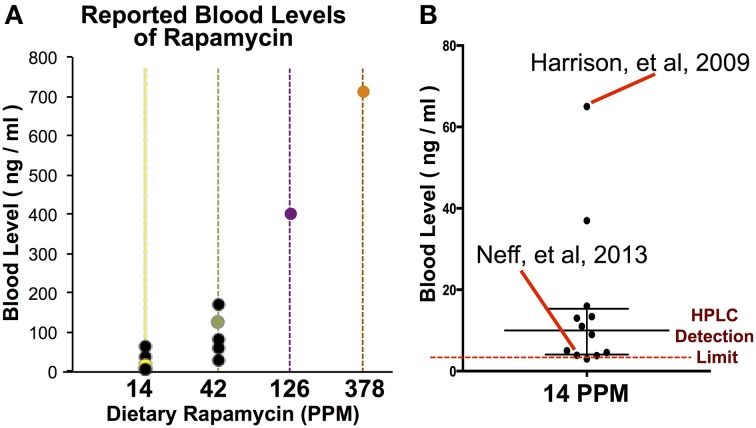
**A comparison of rapamycin blood levels in studies using dietary rapamycin treatment**. **(A)** Blood levels of rapamycin reported in studies using dietary treatment (black datapoints) and in our study (colored datapoints). **(B)** A comparison of reported blood levels in studies using 14 PPM dietary rapamycin with the initial ITP report (Harrison et al.) and the 2013 report suggesting few benefits to aging parameters (Neff et al.) indicated. Bar and whiskers represent median and interquartile range of published blood levels for 14 PPM rapamycin. Reported limit of detection by HPLC indicated by dashed line.

While the beneficial effects of mTOR inhibition by rapamycin on aging and age-related disease have been reproducibly demonstrated, there has been recent debate over whether the anti-cancer effects of rapamycin may account for the lifespan benefits in the mouse model. Notably, a 2013 paper published by Neff et al. (2013) suggested that the effects of rapamycin on murine lifespan are completely explained by the anti-cancer effects of the drugs, rather than an effect on the aging process. Given our findings that dietary rapamycin at 14 PPM is at the low end of the dose-response curve for each of the phenotypes described here, we decided to examine relationships between treatment dose, serum levels, and phenotypic outcome in published studies that utilize this drug in an attempt to explain the somewhat discordant results in the rapamycin literature (Figure 5, Table 1). An examination of studies involving dietary rapamycin use indicates that the blood levels of rapamycin we observed in our mice are consistent with those reported in other studies using 14 and 42 PPM (Figure 5A). A closer inspection of the 14 PPM dose data reveals that the initial 2009 ITP publication of lifespan extension using microencapsulated rapamycin resulted in higher than average blood serum levels of the drug, while the 2013 Neff et al. paper fell at the far low end of the distribution by blood level (Figure 5B). Critically, there is a greater than 10-fold difference in reported blood levels of rapamycin when comparing the 2009 ITP study and the 2013 Neff et al. study, a difference that may largely account for any differences in outcome reported by these groups.

**Table 1:** **A comparison of published studies utilizing dietary encapsulated rapamycin**.

**Dose**	**Blood levels**	**Strain**	**Primary outcome**	**Study**
4.7, 14, 42 PPM	Female: 7, 16, 80 ng/ml Male: 6, 9, 23 ng/ml	UM-HET3	Aging	Miller et al., 2014
14 PPM	Female: ~5 ng/ml Male: ~3 ng/ml	C57Bl/6J and C57Bl/6NIA	Aging	Fok et al., 2014
14 PPM	3–5 ng/ml	C57Bl/6NIA	Aging	Zhang et al., 2014
14, 42 PPM	37, 170 ng/ml	Apc^MIN^	Cancer	Hasty et al., 2014
14 PPM	10–12 ng/ml	TrJ	Nerve mylenation	Nicks et al., 2014
14 PPM	Females: 3.9 ng/ml Males: 3. 8 ng/ml	Rb1+/−	Cancer	Livi et al., 2013
14 PPM	4.57 ng/ml (*n* = 3 mice)	C57Bl/6J	Cancer	Neff et al., 2013
4.7, 14, and 42 PPM	6.5, 13.4, and 57.5 ng/ml	UM-HET3	Aging	Wilkinson et al., 2012
**14 PPM**	**60–70 ng/ml**	**UM-HET3**	**Aging**	**Harrison et al., 2009**
Oral nanoparticles	>3000 ng/ml, no toxicity observed	CD1	Cancer	Bisht et al., 2008
14, 42, 126, and 378 PPM	13, 125, 402, 710 ng/ml	C57Bl/6NIA	Developmental weight, disease	Data here

*The original publication demonstrating murine lifespan extension by microencapsulated rapamycin in bold. Bisht et al., study provided for reference as the highest level of rapamycin reported in mice and no overt toxicity was observed. UM-HET3—genetically heterogenous mice produced from a four-way cross. C57Bl/6 substrain (National Institute on Aging, NIA, vs. Jackson laboratory, J) as indicated*.

## Discussion

Here we present a dose-response profile for dietary rapamycin in a mouse model. Our observations indicate that the commonly used 14 PPM dose is significantly lower than necessary to observe robust effects on developmental weight gain and attenuate disease in the *Ndufs4* KO mouse model of Leigh Syndrome. This data demonstrate that dietary rapamycin at doses much higher than previously tested in normative aging are not only well tolerated, but are also necessary to significantly impact multiple physiological outcomes, raising the possibility that doses higher than 14 PPM may have a more robust impact on longevity and healthspan in normal aging. The standard dose of 14 PPM is at the threshold of detectable effects, indicating it may be at the border of clinical significance. Suboptimal dosing may explain the results of studies that failed to observe significant effects of 14 PPM rapamycin on some parameters of aging (Neff et al., 2013). Rapamycin may be effective in cancer inhibition at doses lower than that necessary for robust inhibition of normal mTOR signaling, and rapamycin diet at the standard ITP dose of 14 PPM may have robust anti-cancer effects with only a relatively modest influence on other age-related pathologies. We suspect that the high doses tested here are likely to provide more potent longevity and healthspan promoting effect than previously reported.

Maintaining high levels of circulating rapamycin also appears necessary to significantly attenuate mitochondrial dysfunction. We previously found that every-other-day injection of 8 mg/kg rapamycin results in only a modest attenuation of disease in the *Ndufs4* KO model while daily injection provides a strong positive effect. A similar response was also seen in the *Lmna* KO mouse model (Ramos et al., 2012). High-dose injection provided the greatest benefit to survival in the *Ndufs4* KO model while oral rapamycin at 378 PPM had the strongest impact on developmental weight gain (Figures 3C,D); the bolus provided by injection provides an enhanced benefit over steady dietary delivery in the mitochondrial disease model, perhaps by overcoming blood-brain barrier. Daily IP injection of rapamycin at 8 mg/kg alters neural activity associated with aging (Yang et al., 2012) and robustly reduces whole brain levels of phospho-s6, an indicator of mTOR activity, in *Ndufs4* KO and WT animals (Johnson et al., 2013c). While the importance of mTOR signaling in brain vs. peripheral tissues in aging and Leigh syndrome is not yet known, these reports demonstrate that circulating levels of rapamycin achieved by daily IP injection are sufficient to reduce mTOR signaling in the central nervous system. Effective inhibition of mTOR in the brain may be necessary for the full benefits of rapamycin in both *Ndufs4* KO mice and in normative aging.

It is worth noting that the use of high dose dietary rapamycin in long-term intervention studies in mice will be restricted by cost, as it is currently only available from a single supplier and this dose is greater than 1000 times more expensive than delivery by injection. 378 PPM dietary rapamycin (378 ug rapamycin per gram-food) is 27 times the dose used by the ITP, while IP injection requires only 8 ug un-encapsulated rapamycin per gram-mouse weight to achieve comparable circulating levels. Alternative methods for delivery of oral rapamycin in mouse chow or the development of stable rapalogs will greatly accelerate efforts to study the effects of high-dose rapamycin in long-term treatment paradigms.

We recognize that the use of high-dose rapamycin raises additional concerns regarding potential side effects. It is important to note that we observed beneficial effects of rapamycin in the mitochondrial disease model when initiated after developmental weight gain—this indicates that the effects of rapamycin treatment on disease in this setting can be at least partially uncoupled from the off-target effects on weight. Conversely, in the setting of a disease with 100% mortality early in life this off-target effect may be seen as tolerable. This will be an important consideration if rapamycin is brought to clinical trials for Leigh Syndrome.

Even at the ITP dosage of 14 PPM, sustained periods of treatment may result in abnormal insulin and glucose responses reminiscent of diabetes (Lamming et al., 2012). However, while mice treated chronically with rapamycin show abnormalities in the context of a glucose tolerance test, this does not equate to a pathogenic state and it remains unclear whether these animals actually have any deficiency in glucose homeostasis under physiologically relevant conditions. Intolerance in this assay may reflect an altered metabolic state wherein metabolism is shifted away from glucose utilization, a state of “starvation diabetes” (Blagosklonny, 2011), where animals are not physiologically primed to deal with a sudden, non-physiological increase in blood glucose levels.

Consistent with this, unpublished data from our laboratory indicates that 90 days of IP injection of 8 mg/kg rapamycin in adult mice does not significantly alter circulating glucose levels in the blood (data not shown). While more work is needed to define the physiological effects of high-dose rapamycin and the true relevance of observed metabolic shifts, our data clearly demonstrate that chronic, very high dose rapamycin has no overt toxicity in mice, and any abnormal physiological findings must be considered in the context of the known benefits of mTOR inhibition in aging or disease paradigms. Furthermore, while it was demonstrated that a modest extension of lifespan in female mice can be achieved in the context of mTORC1 reduction alone, the importance of mTORC2 in the beneficial effects of rapamyicn remains to be determined. The relative roles of mTORC1 and mTORC2 may partially explain the dosage-requirements demonstrated by our work, in addition to the pharmacokinetic properties and tissue specific effects of rapamycin, and will require further study.

### Conflict of interest statement

The authors declare that the research was conducted in the absence of any commercial or financial relationships that could be construed as a potential conflict of interest.

## References

[B1] Anderson S. L. Chung W. K. Frezzo J. Papp J. C. Ekstein J. DiMauro S.. (2008). A novel mutation in NDUFS4 causes Leigh syndrome in an Ashkenazi Jewish family. J. Inherit. Metab. Dis. 31 (Suppl. 2), S461–S467. 10.1007/s10545-008-1049-9 19107570

[B2] Anisimov V. N. Zabezhinski M. A. Popovich I. G. Piskunova T. S. Semenchenko A. V. Tyndyk M. L.. (2011). Rapamycin increases lifespan and inhibits spontaneous tumorigenesis in inbred female mice. Cell Cycle 10, 4230–4236. 10.4161/cc.10.24.18486 22107964

[B3] Bisht S. Feldmann G. Koorstra J. B. M. Mullendore M. Alvarez H. Karikari C.. (2008). *In vivo* characterization of a polymeric nanoparticle platform with potential oral drug delivery capabilities. Mol. Cancer Ther. 7, 3878–3888. 10.1158/1535-7163.MCT-08-0476 19074860 PMC4517597

[B4] Blagosklonny M. V. (2011). Rapamycin-induced glucose intolerance: hunger or starvation diabetes. Cell Cycle 10, 4217–4224. 10.4161/cc.10.24.18595 22157190

[B5] Blagosklonny M. V. (2013). Rapamycin extends life- and health span because it slows aging. Aging 5, 592–598. Available online at: http://www.impactaging.com/papers/v5/n8/pdf/100591.pdf 23934728 10.18632/aging.100591 PMC3796212

[B6] Budde S. M. van den Heuvel L. P. Janssen A. J. Smeets R. J. Buskens C. A. DeMeirleir L.. (2000). Combined enzymatic complex I and III deficiency associated with mutations in the nuclear encoded NDUFS4 gene. Biochem. Biophys. Res. Commun. 275, 63–68. 10.1006/bbrc.2000.3257 10944442

[B7] Budde S. M. S. van den Heuvel L. P. W. J. Smeets R. J. P. Skladal D. Mayr J. A. Boelen C.. (2003). Clinical heterogeneity in patients with mutations in the NDUFS4 gene of mitochondrial complex I. J. Inheri. Metabo. Dis. 26, 813–815. 10.1023/B:BOLI.0000010003.14113.af 14765537

[B8] Budde S. M. S. van den Heuvel L. P. W. J. Smeitink J. A. M. (2002). The human complex I NDUFS4 subunit: from gene structure to function and pathology. Mitochondrion 2, 109–115. 10.1016/S1567-7249(02)00035-1 16120313

[B9] Cheng T. C. Beamer W. G. Phillips J. A. Bartke A. Mallonee R. L. Dowling C. (1983). Etiology of growth hormone deficiency in little, Ames, and Snell dwarf mice. Endocrinology 113, 1669–1678. 10.1210/endo-113-5-1669 6194978

[B10] Choi K. M. Lee H. L. Kwon Y. Y. Kang M. S. Lee S. K. Lee C. K. (2013). Enhancement of mitochondrial function correlates with the extension of lifespan by caloric restriction and caloric restriction mimetics in yeast. Biochem. Biophys. Res. Commun. 441, 236–242. 10.1016/j.bbrc.2013.10.049 24141116

[B11] Darin N. Oldfors A. Moslemi A. R. Holme E. Tulinius M. (2001). The incidence of mitochondrial encephalomyopathies in childhood: clinical features and morphological, biochemical, and DNA anbormalities. Ann. Neurol. 49, 377–383. 10.1002/ana.75 11261513

[B12] Fok W. C. Chen Y. Bokov A. Zhang Y. Salmon A. B. Diaz V.. (2014). Mice fed rapamycin have an increase in lifespan associated with major changes in the liver transcriptome. PLoS ONE 9:e83988. 10.1371/journal.pone.0083988 24409289 PMC3883653

[B13] Ha C. W. Huh W. K. (2011). Rapamycin increases rDNA stability by enhancing association of Sir2 with rDNA in Saccharomyces cerevisiae. Nucleic Acids Res. 39, 1336–1350. 10.1093/nar/gkq895 20947565 PMC3045593

[B14] Harrison D. E. Strong R. Sharp Z. D. Nelson J. F. Astle C. M. Flurkey K.. (2009). Rapamycin fed late in life extends lifespan in genetically heterogeneous mice. Nature 460, 392–395. 10.1038/nature08221 19587680 PMC2786175

[B15] Hasty P. Livi C. B. Dodds S. G. Jones D. Strong R. Javors M.. (2014). eRapa restores a normal life span in a FAP mouse model. Cancer Prev. Res. 7, 169–178. 10.1158/1940-6207.CAPR-13-0299 24282255 PMC4058993

[B16] Johnson S. C. Martin G. M. Rabinovitch P. S. Kaeberlein M. (2013a). Preserving youth: does rapamycin deliver? Science Trans. Med. 5, 211fs240. 10.1126/scitranslmed.3007316 24225941 PMC4019780

[B17] Johnson S. C. Rabinovitch P. S. Kaeberlein M. (2013b). mTOR is a key modulator of ageing and age-related disease. Nature 493, 338–345. 10.1038/nature11861 23325216 PMC3687363

[B18] Johnson S. C. Yanos M. E. Kayser E. B. Quintana A. Sangesland M. Castanza A.. (2013c). mTOR inhibition alleviates mitochondrial disease in a mouse model of Leigh syndrome. Science 342, 1524–1528. 10.1126/science.1244360 24231806 PMC4055856

[B19] Kaeberlein M. (2013). mTOR Inhibition: from aging to Autism and beyond. Scientifica 2013:849186. 10.1155/2013/849186 24379984 PMC3860151

[B20] Kruse S. E. Watt W. C. Marcinek D. J. Kapur R. P. Schenkman K. A.. (2008). Mice with mitochondrial complex I deficiency develop a fatal encephalomyopathy. Cell Metab. 7, 312–320. 10.1016/j.cmet.2008.02.004 18396137 PMC2593686

[B21] Lamming D. W. Ye L. Katajisto P. Goncalves M. D. Saitoh M. Stevens D. M.. (2012). Rapamycin-induced insulin resistance is mediated by mTORC2 loss and uncoupled from longevity. Science 335, 1638–1643. 10.1126/science.1215135 22461615 PMC3324089

[B22] Livi C. B. Hardman R. L. Christy B. A. Dodds S. G. Jones D. Williams C.. (2013). Rapamycin extends life span of Rb1^+/−^ mice by inhibiting neuroendocrine tumors. Aging 5, 100–110. Available online at: http://www.impactaging.com/papers/v5/n2/full/100533.html 23454836 10.18632/aging.100533 PMC3616197

[B23] Miller R. A. Harrison D. E. Astle C. M. Baur J. A. Boyd A. R. de Cabo R.. (2011). Rapamycin, but not resveratrol or simvastatin, extends life span of genetically heterogeneous mice. J. Gerontol. 66, 191–201. 10.1093/gerona/glq178 20974732 PMC3021372

[B24] Miller R. A. Harrison D. E. Astle C. M. Fernandez E. Flurkey K. Han M.. (2014). Rapamycin-mediated lifespan increase in mice is dose and sex dependent and metabolically distinct from dietary restriction. Aging cell 13, 468–477. 10.1111/acel.12194 24341993 PMC4032600

[B25] Neff F. Flores-Dominguez D. Ryan D. P. Horsch M. Schröder S. Adler T.. (2013). Rapamycin extends murine lifespan but has limited effects on aging. J. Clin. Invest. 123, 3272–3291. 10.1172/JCI67674 23863708 PMC3726163

[B26] Nicks J. Lee S. Harris A. Falk D. J. Todd A. G. Arrendondo K.. (2014). Rapamycin improves peripheral nerve myelination while it fails to benefit neuromuscular performance in neuropathic mice. Neurobiol. Disease 70, 224–236. 10.1016/j.nbd.2014.06.023 25014022 PMC4532623

[B27] Partridge L. Alic N. Bjedov I. Piper M. D. W. (2011). Ageing in *Drosophila*: the role of the insulin/Igf and TOR signalling network. Exp. Gerontol. 46, 376–381. 10.1016/j.exger.2010.09.003 20849947 PMC3087113

[B28] Quintana A. Kruse S. E. Kapur R. P. Sanz E. Palmiter R. D. (2010). Complex I deficiency due to loss of *Ndufs4* in the brain results in progressive encephalopathy resembling Leigh syndrome. Proc. Natl. Acad. Sci. U.S.A. 107, 10996–11001. 10.1073/pnas.1006214107 20534480 PMC2890717

[B29] Ramos F. J. Chen S. C. Garelick M. G. Dai D. F. Liao C. Y. Schreiber K. H.. (2012). Rapamycin reverses elevated mTORC1 signaling in lamin A/C-deficient mice, rescues cardiac and skeletal muscle function, and extends survival. Sci. Transl. Med. 4, 144ra103. 10.1126/scitranslmed.3003802 22837538 PMC3613228

[B30] Spong A. Bartke A. (2012). Rapamycin slows aging in mice. Cell Cycle 11, 845. 10.4161/cc.11.5.19607 22356747

[B31] Wilkinson J. E. Burmeister L. Brooks S. V. Chan C. Friedline S. Harrison D. E.. (2012). Rapamycin slows aging in mice. Aging Cell 11, 675–682. 10.1111/j.1474-9726.2012.00832.x 22587563 PMC3434687

[B32] Yang S. B. Tien A. C. Boddupalli G. Xu A. W. Jan Y. N. Jan L. Y. (2012). Rapamycin ameliorates age-dependent obesity associated with increased mTOR signaling in hypothalamic POMC neurons. Neuron 75, 425–436. 10.1016/j.neuron.2012.03.043 22884327 PMC3467009

[B33] Zhang Y. Bokov A. Gelfond J. Soto V. Ikeno Y. Hubbard G.. (2014). Rapamycin extends life and health in C57BL/6 mice. J. Gerontol. A Biol. Sci. Med. Sci. 69, 119–130. 10.1093/gerona/glt056 23682161 PMC4038246

